# Determination of promising inhibitors for N-SH2 domain of SHP2 tyrosine phosphatase: an in silico study

**DOI:** 10.1007/s11030-024-10880-2

**Published:** 2024-05-13

**Authors:** Emel Başak Gencer Akçok, Hüseyin Güner, İsmail Akçok

**Affiliations:** 1 Department of Molecular Biology and Genetics, Faculty of Life and Natural Sciences, Abdullah Gül University, 38080 Kayseri, Türkiye; 2 Department of Bioengineering, Faculty of Life and Natural Sciences, Abdullah Gül University, 38080 Kayseri, Türkiye; 3 Izmir International Biomedicine and Genome Institute, Dokuz Eylul University, 35340 Balçova, İzmir Türkiye; 4 Izmir Biomedicine and Genome Center (IBG), 35340 Balçova, İzmir Türkiye

**Keywords:** Molecular docking, Molecular dynamics (MD), In silico, SHP2 phosphatase, SH2 domain

## Abstract

There are many genes that produce proteins related to diseases and these proteins can be targeted with drugs as a potential therapeutic approach. Recent advancement in drug discovery techniques have created new opportunities for treating variety of diseases by targeting disease-related proteins. Structure-based drug discovery is a faster and more cost-effective approach than traditional methods. SHP2 phosphatase, encoded by the *PTPN11* gene, has been the focus of much attention due to its involvement in many types of diseases. The biological function of SHP2 is enabled mostly by protein–protein interaction through its SH2 domains. In this study, we report the identification of a potential small molecule inhibitor for the N-SH2 domain of SHP2 by structure-based drug discovery approach. We utilized molecular docking studies, followed by molecular dynamics simulations and MM/PBSA calculations, to analyze compounds retrieved from the Broad's Drug Repurposing Hub and ZINC15 databases. We selected 10 hit compounds with the best docking scores from the libraries and examined their binding properties in the N-SH2 domain. We found that compound CID 60838 (Irinotecan) was the most suitable compound with a binding free energy value of − 64.45 kcal/mol and significant interactions with the target residues in the domain.

**Graphical abstract:**

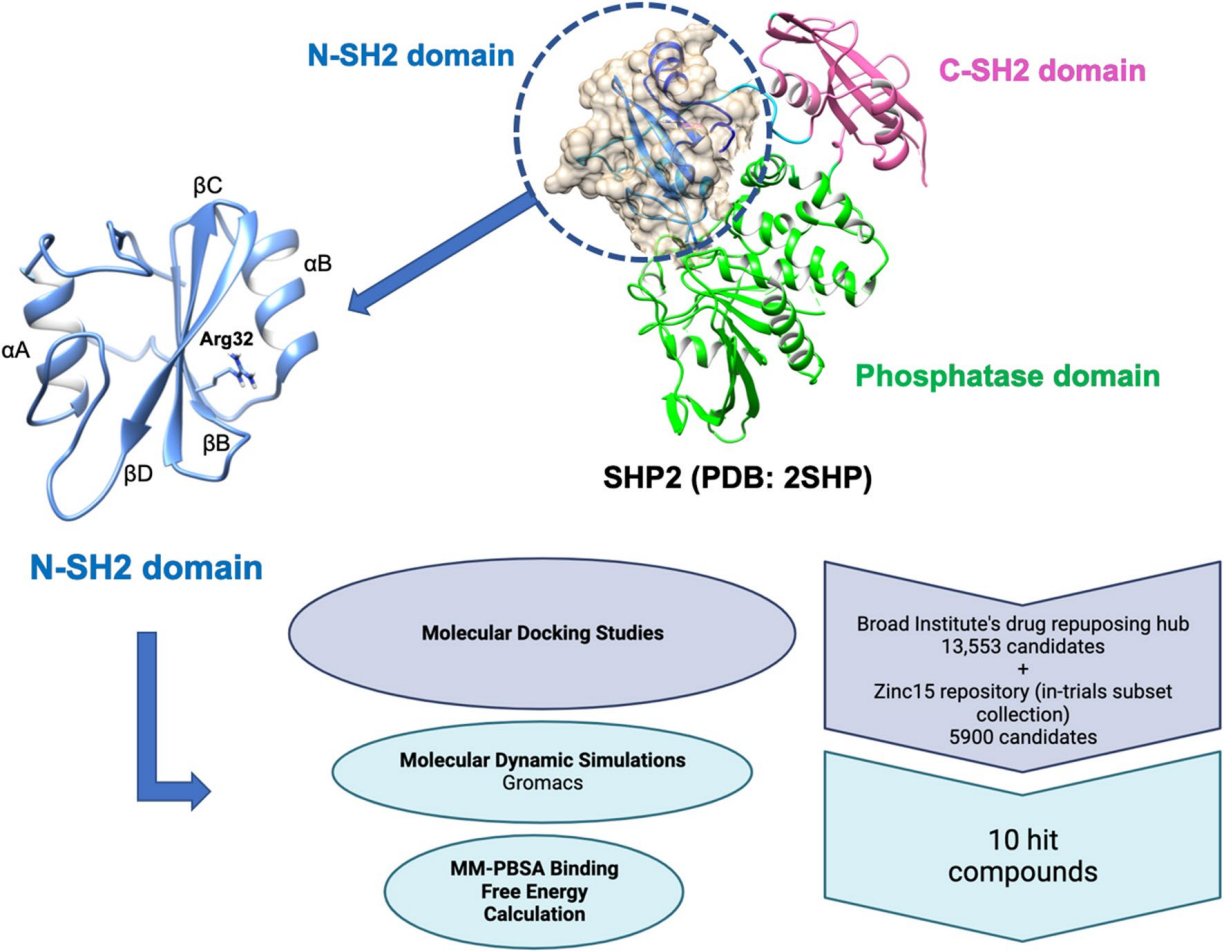

**Supplementary Information:**

The online version contains supplementary material available at 10.1007/s11030-024-10880-2.

## Introduction

Specific protein–protein interaction is very important for the organization of intracellular signaling cascades and these interactions are mediated by protein domains [1]. The protein-mediated signaling processes sometimes require post-translational modifications and among these post-translational modifications, phosphorylation of specific residues such as tyrosine, serine, and threonine has great importance [2]. Tyrosine phosphorylation essentially has a critical importance for mammalian cells and studies demonstrated that it has particular importance in differentiation, proliferation, apoptosis and migration [3]. Tyrosine phosphorylation is mediated by proteins called protein tyrosine kinases and they are often called as “writers”. The phosphorylated tyrosine residues are recognized by the proteins via their SH2 (Src Homology 2) domains. There are also proteins that remove the phosphate groups which are protein tyrosine phosphatases, and they are often referred as “erasers” [4].

SH2 domains are noncatalytic and highly conserved modules that contain approximately 100 amino acids and their structure is composed of a central antiparallel β-sheet flanked by two α-helices. The secondary structures, α-helices and β-sheets are connected by loop structures which are not folded and flexible regions of the target protein. Although the secondary structures are conserved in SH2 family of proteins, the loop sequences are not conserved. Protein phosphorylation is a fundamental post-translational modification that regulates many important signal transduction pathways, and the tyrosine phosphorylation has a particular importance in cell signaling, cell proliferation, growth, differentiation, cell survival, and even cell death. The protein interaction occurs through the interaction of phosphotyrosine and SH2 domains. Therefore, SH2 domains are very important regulators by maintaining specific phosphotyrosine signaling [4]. There are 121 SH2 domains in 111 different proteins [5]. SH2 domains recognize the phosphorylated tyrosine residues and through this binding, they form multi-protein complexes [6]. By facilitating the protein–protein interactions, SH2 domains have a critical role in regulation of different essential cellular functions [7]. The specific recognition and binding to the phosphorylated tyrosine residues on target proteins are enabled by a specific amino acid sequence on SH2 domain. This conserved motif, G(S/T)FLVR(E/D)S, contains a positively charged arginine residue which is critical for binding to the negatively charged phosphate group on tyrosine residues [8, 9]. This conserved arginine residue on the strand βB plays a central role in forming bi-dentate hydrogen bonds with the phosphate moiety [10].

**Fig. 1:**
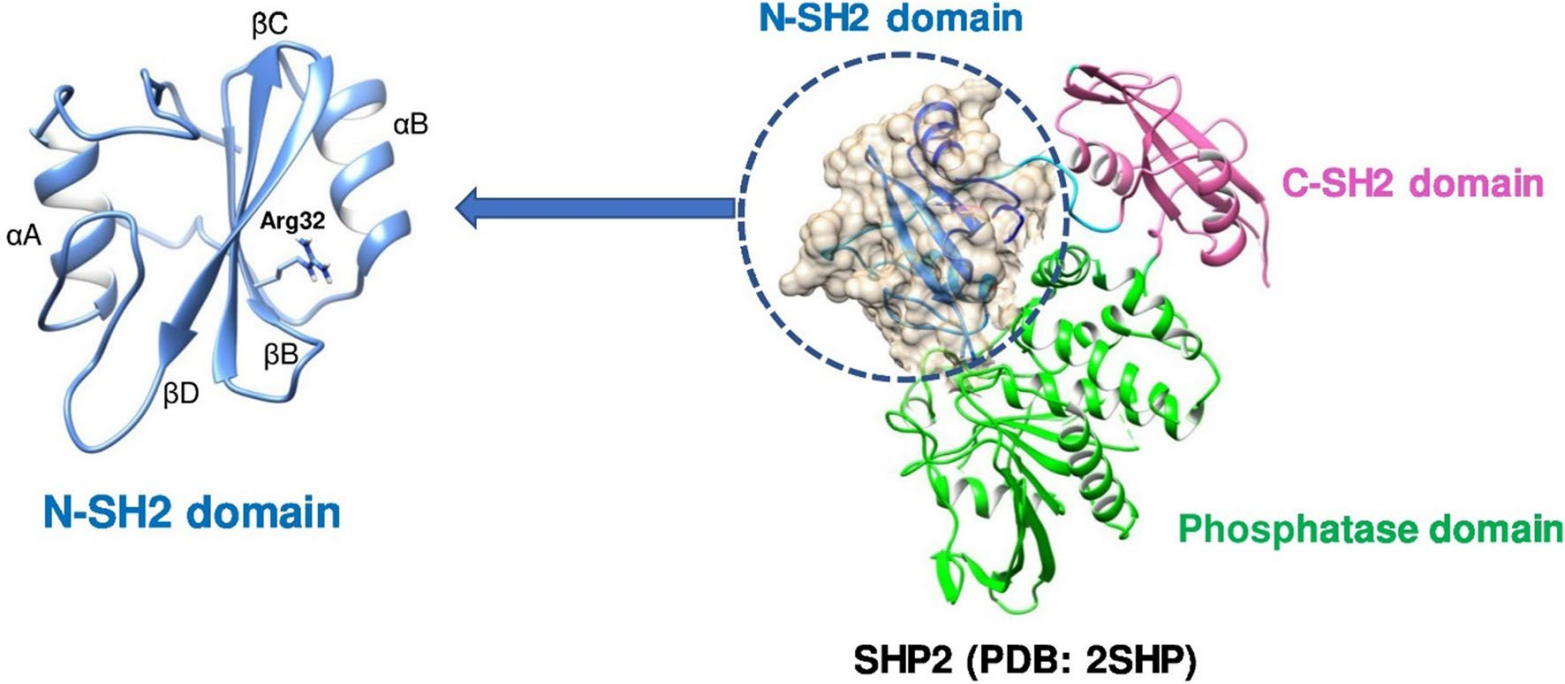
Cartoon representation of SHP2 full length (PDB ID: 2SHP) with N-SH2 binding site

SH2 domain-containing phosphatase 2 (SHP2), which is encoded by the protein tyrosine phosphatase non-receptor type 11 (*PTPN11*) gene, is a tyrosine phosphatase that is composed of two SH2 domains at the amino terminus of the protein tyrosine phosphatase (PTP) domain which is located at the carboxy terminus (Fig. 1) [11]. Under normal unstimulated conditions, SHP2 possesses a closed conformation in which the N-SH2 binds to the PTP domain and thus remains inactive. When the SH2 domains encounter a target protein that has phosphorylated tyrosine residues, the conformation of the protein is opened and the phosphatase becomes active [12].

SH2 domains of SHP2 have functional importance in several diseases [13]. SHP2 phosphatase is an important regulator of cytokine, growth factor, and integrin signaling and thus has crucial importance in cell survival, proliferation, and differentiation by regulating major signaling pathways [14]. Under normal unstimulated conditions, SHP2 possesses a closed conformation in which the N-SH2 binds to the PTP domain and thus remains inactive. However, when the SH2 domains encounter a target protein that has phosphorylated tyrosine residues, the conformation of the SHP2 protein is opened and the phosphatase becomes constitutively active [12]. There are mutations that are identified on *PTPN11* gene which encodes the SHP2 phosphatase, identified in several diseases, as such leukemia, solid tumors, Noonan syndrome and LEOPARD syndrome [13]. SHP2 was the first phosphatase that was discovered to be oncogenic [15]. Gain-of-function *PTPN11* mutations are the cause of more than 30% of juvenile myelomonocytic leukemia (JMML) and can be also found in other childhood malignancies [16, 17]. Moreover, SHP2 has also a critical role in receptor tyrosine kinase-driven cancers [18]. Missense mutations of *PTPN11* are found in more than 50% of the Noonan syndrome cases, which is a genetic disorder resulting from hyperactivation of the Ras-MAPK pathway [19, 20]. These missense mutations are gain-of-function mutations that keep phosphatase active [21]. For all these involvements of SHP2 in various disorders, it is important to target SHP2 phosphatase. Although there are some compounds targeting SHP2, genomic screens demonstrate that resistance to SHP2 inhibition is conferred [22]. Considering the role of SHP2 in a variety of diseases, new candidates are required to be identified. Computational methods are useful tools to identify novel hit compounds for a target at the atomic level for drug repurposing.

In the current study, we aimed to find the potential inhibitor that targets the N-SH2 domain of the SHP2 protein. For this purpose, a virtual screening protocol that consists of molecular docking, protein–ligand interaction, molecular dynamics (MD) simulations, and molecular mechanics Poisson–Boltzmann surface area (MM/PBSA) calculations were applied to Broad Repurposing Hub compounds, and in-trial subsets from ZINC15 library. The interactions of ligands with key residues were also shown and identified.

## Methodology

### Protein crystal structure preparation and ligand preparation

SHP-2 (PDB ID: 2SHP) [23] was selected as the target protein’s X-ray crystallography model file and retrieved from the RCSB Protein Data Bank (www.rcsb.org). The protein was prepared for docking studies using PDBFixer (https://github.com/openmm/pdbfixer) [24]. It was cleaned off the existing water molecules and the missing residues and hydrogens were added to the protein. The Fpocket tool [25] was used to inspect and identify potentially druggable pockets. Pocket 15 was chosen as the potential protein–ligand binding site. PyMol software was used to visually inspect the druggable pockets and identify the binding area encompassing the biologically essential binding domain of N-SH2.

The Broad Institute’s drug repurposing hub (https://www.broadinstitute.org/drug-repurposing-hub) provides a list of chemicals to screen potential drugs. Their repository contains a collection of 13553 FDA-approved drugs, clinical trials, and pre-clinical compounds. The structure files were downloaded of matching items of selected compounds from PubChem. A similar drug repurposing list of 5900 substances from the Zinc15 repository was also retrieved by browsing the in-trials subset collection [26]. All available structure files were downloaded accordingly. Because the three-dimensional structural files for some of the ligands were not available, they were processed using RDKit [27] and thus the hydrogens were added, and the energies of the 3D structures of each ligand in the screening library were minimized before docking.

### Molecular docking studies

Smina [28], a variant of Autodock Vina [29], was used in molecular docking studies. The grid box of a rectangular parallelogram was selected with the coordinates (− 2.5, − 38.8, 39.3) and axis sizes (14.0, 14.6, 19.3) plus 4 Angstrom (Å) on each axis as the search space for the best binding poses. The exhaustiveness parameter was set as 16 and the seed was selected as 43.

### MD simulation

MD simulations were carried out using Gromacs ver. 2021.03 [30]. The protein structure was cleaned and prepared for running simulations processing the script provided by the Chimera package. OPLS-AA/M as the force field and SPC216 as the explicit water models were selected to describe the ensemble. Ligand topologies and their force field parameter files for the force field selected were calculated by the LigParGen [31] command-line tool. (Please see the detailed MD methodology in Supporting Information).

### MM/PBSA binding free energy calculation

The intensity of the binding free energy between ligands and receptors was estimated using MM/PBSA binding free energy calculations [32]. Sets of 200 configurations for each system were obtained as 1 nanosecond (ns) spaced snapshots, obtained directly from the molecular dynamics trajectories. The calculations were carried out using the g_mmpbsa GROMACS-compatible free energy program [33] with a grid space of 0.5 Å, salt concentration of 0.150 M, solute dielectric constant of 2 and employing the solvent accessible surface area (SASA) as estimate of the nonpolar solvation energy.

## Results and discussion

### Selection of target protein and identification of drug candidates

In this work, we target the specific recognition and binding region of the N-SH2 domain of SHP2, which has a conserved motif of G(S/T)FLVR(E/D)S. The specific positively charged arginine residue (Arg32) which is critical for binding to the negatively charged phosphate group on tyrosine residues was targeted. Because of the importance of this residue in protein–protein interaction and SHP2 signaling, targeting Arg32 is the main motivation of this study. The most conserved residues reside on the βB strand, the FLVR motif is present with the conserved arginine residue that plays the central role in forming an interaction with the phosphate group of phosphotyrosine [34]. Moreover, thermodynamic studies have established that the ionic interaction between Arg βB5 and pTyr is crucial for the affinity, providing more than half of the total binding energy [35]. Thus, we targeted the phosphotyrosine binding site of SHP2 (N-SH2 domain) for in silico screening with the goal of finding molecules that inhibit the interaction between N-SH2 and pTyr.

### Molecular docking analysis of drugs against N-SH2 domain of 2SHP

Molecular docking studies have become essential for the discovery of novel drug candidates with the advancement of computational tools. The experimental binding mechanism and affinity were predicted by this method and it provides invaluable information about the possible interactions.

**Fig. 2:**
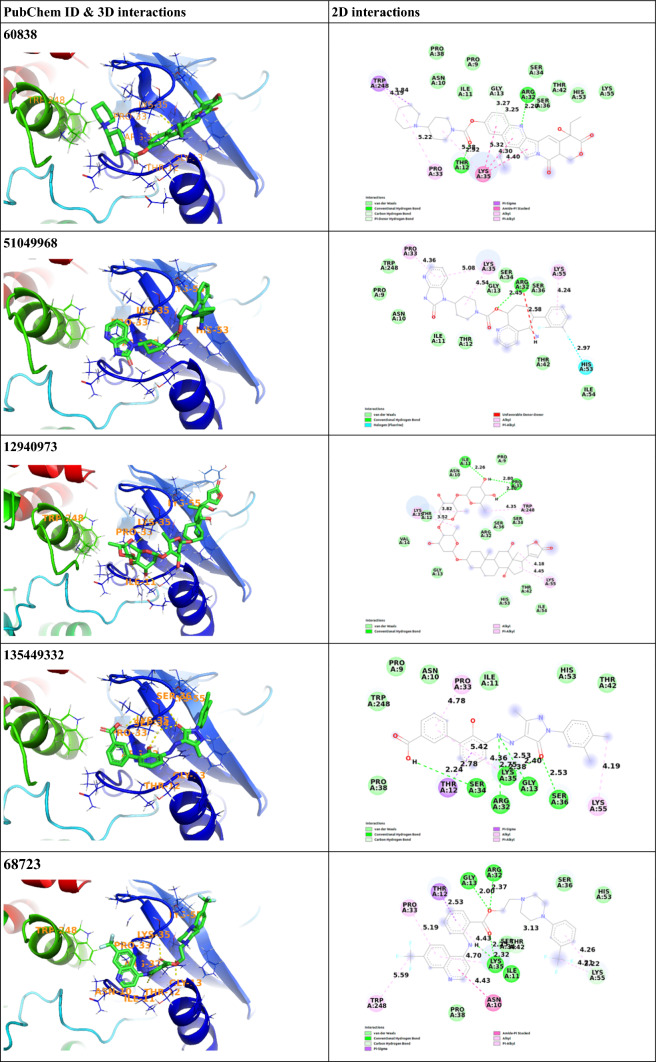

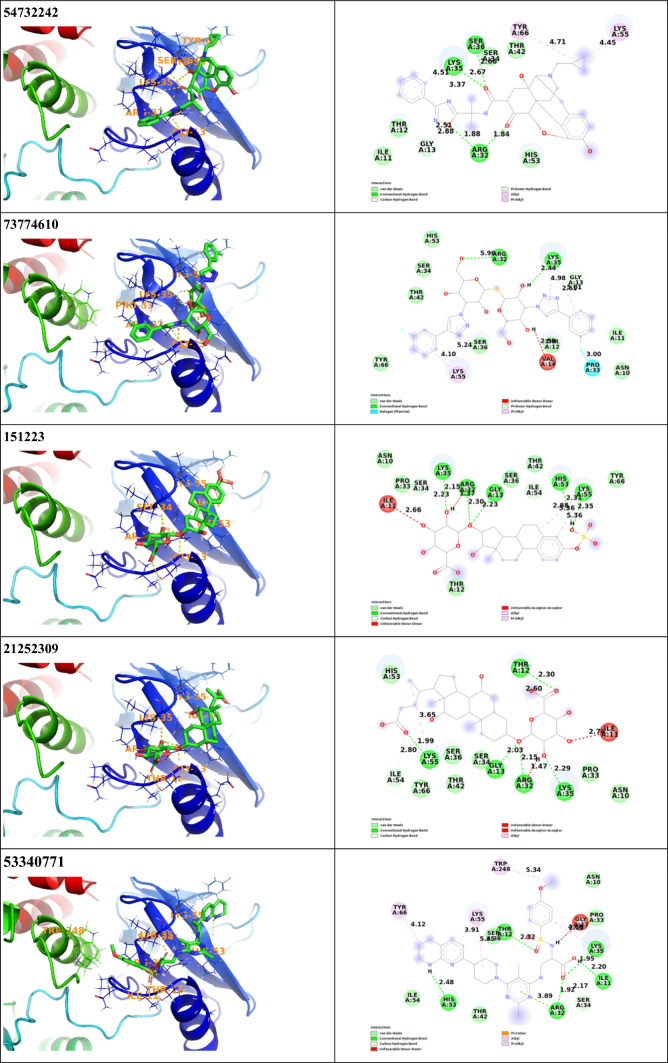
Binding modes of top ten candidates against N-SH2 domain of 2SHP

The molecular docking study of the compounds from The Broad Institute’s FDA-approved drug and ZINC15 in-trials subset was carried out SMINA docking program to get the lowest energy conformers of the compounds at the binding site of the N-SH2 domain of SHP2 protein. At this stage, all known kinase inhibitors were eliminated from the list as they are known to bind to binding site which is not targeted in the current study. The molecular docking data of the compounds were collected and the best ten candidates were chosen for further studies. All docking scores, main interacting residues of the top ten candidates and interaction types of these molecules are listed in Table 1 and demonstrated in Fig. 2. The best docking score against 2SHP was achieved by Irinotecan (CID 60838) with a value of − 9.3 kcal/mol, followed by Rimegepant (CID 51049968) with − 9.2 kcal/mol, CID 12940973 with − 9.1 kcal/mol, Eltrombopag (CID 135449332) with − 9.1 kcal/mol, Antrafenine (CID 68723) with − 8.9 kcal/mol, Naldemedine (CID 54732242) with − 8.8 kcal/mol, CID 73774610 with − 8.7 kcal/mol, Estriol 3-sulfate 16-glucuronide (CID 151223) with − 8.7 kcal/mol, Cholic acid glucuronide (CID 21252309) with − 8.7 kcal/mol, Glpg-0187 (CID 53340771) with − 8.7 kcal/mol respectively. The results show that all these ten molecules make hydrogen bonds with Arg32 as well as other interacting residues such as Thr12, Gly13, Lys35, Lys55 and Ile11. (Table 1).

**Table 1:** Molecular docking results and interaction details of top 10 hit molecules against 2SHP protein

PubChem ID/name	Main interacting residues	Distance (Å)	Interaction types	Binding affinity (kcal/mol)
60838/Irinotecan	Arg32 Thr12 Trp248 Trp248 Lys35 Lys35 Lys35 Pro33 Gly13 Gly13	2.20 2.92 3.84 4.19 4.40 4.30 5.32 5.22 3.27 3.25	Conventional hydrogen bond Conventional hydrogen bond Π—sigma Π—alkyl Π—alkyl Π—alkyl Π—alkyl Alkyl Π donor—hydrogen bond Π donor—hydrogen bond	− 9.3
51049968/Rimegepant	Arg32 Lys35 Lys35 Pro33 Lys55 His53	2.45 4.54 5.08 4.36 4.24 2.97	Conventional hydrogen bond Alkyl Π—alkyl Π—alkyl Π—alkyl Halogen (fluorine)	− 9.2
12940973	Pro33 Pro33 Ile11 Lys35 Lys35 Lys55 Lys55 Trp248	2.20 2.80 2.26 3.82 3.52 4.18 4.45 4.35	Conventional hydrogen bond Conventional hydrogen bond Conventional hydrogen bond Alkyl Alkyl Alkyl Alkyl Π—alkyl	− 9.1
135449332/Eltrombopag	Arg32 Gly13 Ser34 Lys35 Lys35 Ly35 Ser36 Thr12 Pro33 Lys55	2.75 2.53 2.24 2.38 4.36 5.42 2.53 2.78 4.78 4.19	Conventional hydrogen bond Conventional hydrogen bond Conventional hydrogen bond Conventional hydrogen bond Π—alkyl Π—alkyl Conventional hydrogen bond Π—sigma Π—alkyl Alkyl	− 9.1
68723/Antrafenine	Arg32 Gly13 Ile11 Lys35 Lys35 Lys35 Lys55 Lys55 Asn10 Try248 Pro33 Thr12	2.37 2.00 2.32 2.25 4.43 4.70 4.21 4.26 4.43 5.59 5.19 2.53	Conventional hydrogen bond Conventional hydrogen bond Conventional hydrogen bond Conventional hydrogen bond Π—alkyl Π—alkyl Alky Π—alkyl Amide—Π stacking Π—alkyl Π—alkyl Π—sigma	− 8.9
54732242/Naldemedine	Arg32 Arg32 Gly13 Gly13 Lys35 Lys35 Lys35 Ser36 Lys55 Tyr66	1.84 1.88 2.51 2.88 2.67 3.37 4.51 2.66 4.45 4.71	Conventional hydrogen bond Conventional hydrogen bond Π donor–hydrogen bond Carbon hydrogen bond Conventional hydrogen bond Π donor–hydrogen bond Π—alkyl Conventional hydrogen bond Alkyl Alkyl	− 8.8
73774610	Arg32 Lys35 Lys35 Lys35 Gly13 Pro33 Lys55 Lys55	2.96 2.44 4.98 5.01 2.61 3.00 4.10 5.24	Conventional hydrogen bond Conventional hydrogen bond Π—alkyl Π—alkyl Π donor–hydrogen bond Halogen (fluorine) Π—alkyl Π—alkyl	− 8.7
151223/Estriol 3-sulfate 16-glucuronide	Arg32 Gly13 Lys35 Ser34 His53 Lys55 Lys55 Lys55 Ile54	2.30 2.23 2.15 2.23 2.31 2.35 5.36 5.36 2.88	Conventional hydrogen bond Conventional hydrogen bond Conventional hydrogen bond Carbon hydrogen bond Conventional hydrogen bond Conventional hydrogen bond Π—alkyl Alkyl Carbon hydrogen bond	− 8.7
21252309/Cholic acid glucuronide	Arg32 Thr12 Thr12 Gly13 Lys35 Lys55 Lys55 Ile54	2.15 2.30 2.60 2.03 2.29 1.99 3.65 2.80	Conventional hydrogen bond Conventional hydrogen bond Carbon hydrogen bond Conventional hydrogen bond Conventional hydrogen bond Conventional hydrogen bond Alkyl Carbon hydrogen bond	− 8.7
53340771/Glpg-0187	Arg32 Arg32 Ser34 Ile11 Lys35 Lys35 Thr12 Trp248 Lys55 Lys55 Tyr66 His53	3.89 1.92 2.17 2.20 1.95 4.60 2.32 5.34 5.35 3.91 4.12 2.48	Π—cation Conventional hydrogen bond Carbon hydrogen bond Conventional hydrogen bond Conventional hydrogen bond Π—alkyl Conventional hydrogen bond Π—alkyl Alkyl Π—alkyl Π—alkyl Conventional hydrogen bond	− 8.7

### MD simulations of selected drugs against N-SH2 domain of 2SHP

MD simulation, which is accepted as the gold standard to test the stability of the complex formed between protein and the leading drug candidates [36], has been performed for the best docking poses of the top ten candidates. For a comprehensive evaluation of the ligand–protein complexes, 300 ns molecular dynamic simulation was performed.

**Fig. 3:**
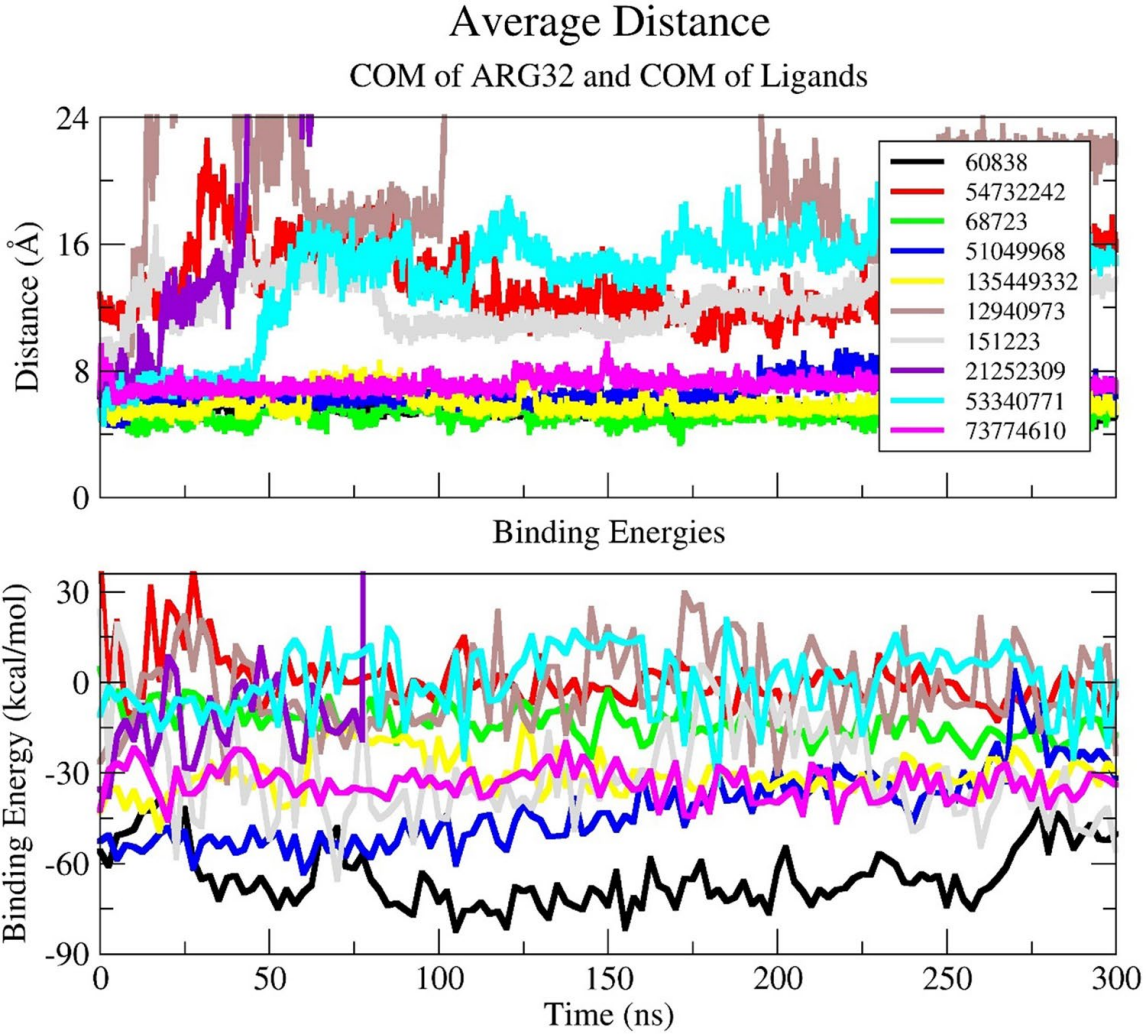
**A** Average distance between COM of Arg32 and COM of the ligands and **B** MM/PBSA binding free energy plots of top ten ligands

There are a lot of data that can be retrieved from MD simulations. In our study, first, we evaluated the distance between the center of mass (COM) of the ligands and the COM of Arg32. As mentioned previously, with its positive charge Arg32 residue is essential for binding tyrosine phosphorylated substrates. Focusing on the distance between this important residue and the corresponding ligands during the simulation provided us an important information about the binding affinity and stability of the complex. By measuring this distance, we could easily determine whether the ligands maintain their positions during the simulation. As shown in Fig. 3A, it is clearly seen that the COM of the ligands CID 12940973, CID 21252309, CID 54732242 move away from Arg32 residue at the beginning of the simulation. The distance between COM of CID 53340771 and Arg32 is maintained as 7 Å until 50 ns, then the distance is calculated as 15 Å approximately. The distance between COM of the ligands CID 68723, CID 60838, CID 51049968, CID 73774610, and CID 135449332 is very close with Arg32 (between 4 and 7 Å), and they retain their position during the simulation.

**Fig. 4:**
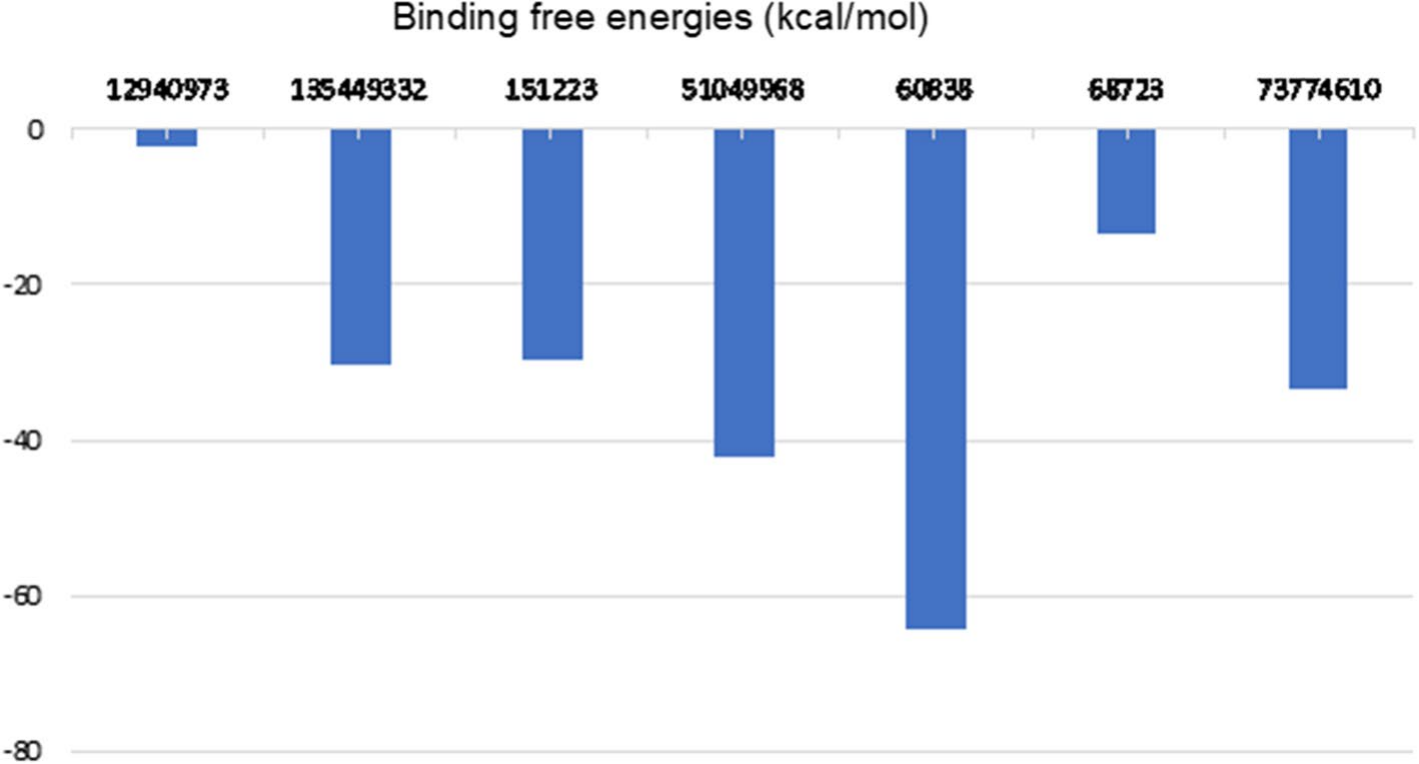
MM/PBSA results for the 2SHP complexed with hit compounds. Only negative binding free energies are listed

Next, the binding free energies of the ligands were calculated by MM/PBSA analysis (Fig. 3B). In Fig. 4, the bar plots clearly demonstrated that 6 ligands among 10 hits have presented good binding free energy. Ligand CID 60838 has shown the best binding free energy with − 64.45 kcal/mol. Ligand CID 51049968 has shown the second-best binding free energy with − 42.13 kcal/mol. The energies of other ligands are listed in Fig. 4 and Table S1. (see Supporting information Table S1). Surprisingly, ligand CID 21252309 has demonstrated huge positive binding energy (1974 kcal/mol). When MM/PBSA calculation energies were checked (see Supporting information Table S2) for this ligand, a very large polar solvation energy was observed. This could be the explanation for such a large positive energy. Although it had a remarkable docking score (− 8.7 kcal/mol), it was observed as the least stable ligand during the MD simulation (see MD movie_supporting information). Besides, the energy calculations table (Supporting information Table S2) shows that ligands CID 12940973, CID 53340771 and CID 54732242 have relatively small van der Waal energy, and high electrostatic energies compared to other ligands. Therefore, these three ligands have exhibited weaker binding energies with − 2.21 kcal/mol, 0.39 kcal/mol, and 0.70 kcal mol, respectively. These results also proved why a long-time MD simulation analysis and MM/PBSA calculations are necessary to decide the stability of the ligand–protein complexes.

**Fig. 5:**
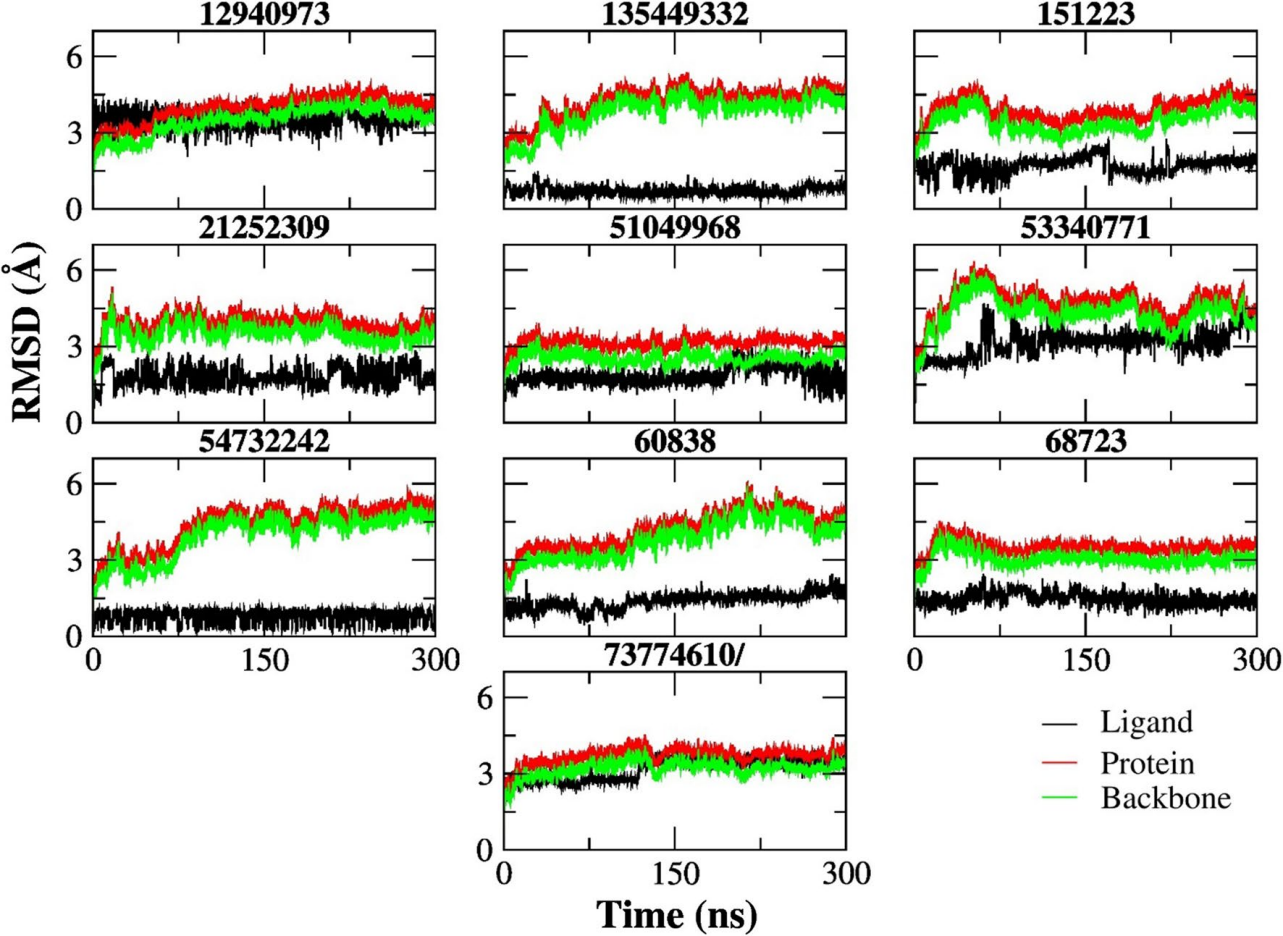
RMSD plots of 10 hit compounds during 300 ns simulations

The root mean square deviation (RMSD) plots of ten hit ligands were created to measure the difference between the protein’s backbone atoms from its initial conformation to its final conformation. The stability of the protein can be determined by the fluctuations observed during the simulations. The smaller the fluctuations, the more stable the protein structure. In Fig. 5, RMSD plots show that the fluctuations of all proteins do not pass beyond 6 Å for the proteins, and 4 Å for the ligands. In the figure, it is clearly seen that the ligands which have closer contact with Arg32 have smaller RMSD values, and show fewer fluctuations during the 300 ns simulations, except for CID 73774610. Analysis of the dynamic behavior of the compounds helped us to understand the stabilization of the ligands in the binding site. Superpositions of the frames of the selected ligands were collected during the MD simulations shown in Fig. S4. These results also reflect the Fig. 5 which shows small RMSD fluctuations for compounds such CID 60838, CID 68723 (see the movies supplied as Supporting Information).

The root mean square fluctuation (RMSF) method predicts the protein’s stability by estimating the average atomic flux of individual amino acids across the simulation period compared to the initial structure. When we examine the top three ligands, it can be easily observed that the fluctuation of amino acids for each protein–ligand complex mostly under 4 Å. These results indicate that the binding of ligands does not affect the stability of the protein. The RMSF plot of top three ligands is given in the supporting information (Fig. S5).

The radius of gyration (*R*g) is also calculated from the MD simulation trajectories, which calculates the RMSD values between the protein center of gravity and the protein residues. When a ligand binds to a protein, there is a conformational change that affects the radius of gyration. Investigation of *R*g determines the compactness of the protein–ligand complex, and gives an idea about the stability of the protein. A small fluctuations in *R*g indicates that the ligand does not change the protein conformation. *R*g analysis of top 10 ligands showed similar *R*g values in the range 2.5 to 2.6 nm. The radii of gyration showed a stable trend in all complexes during the 300 ns simulation (Fig. S6).

**Fig. 6:**
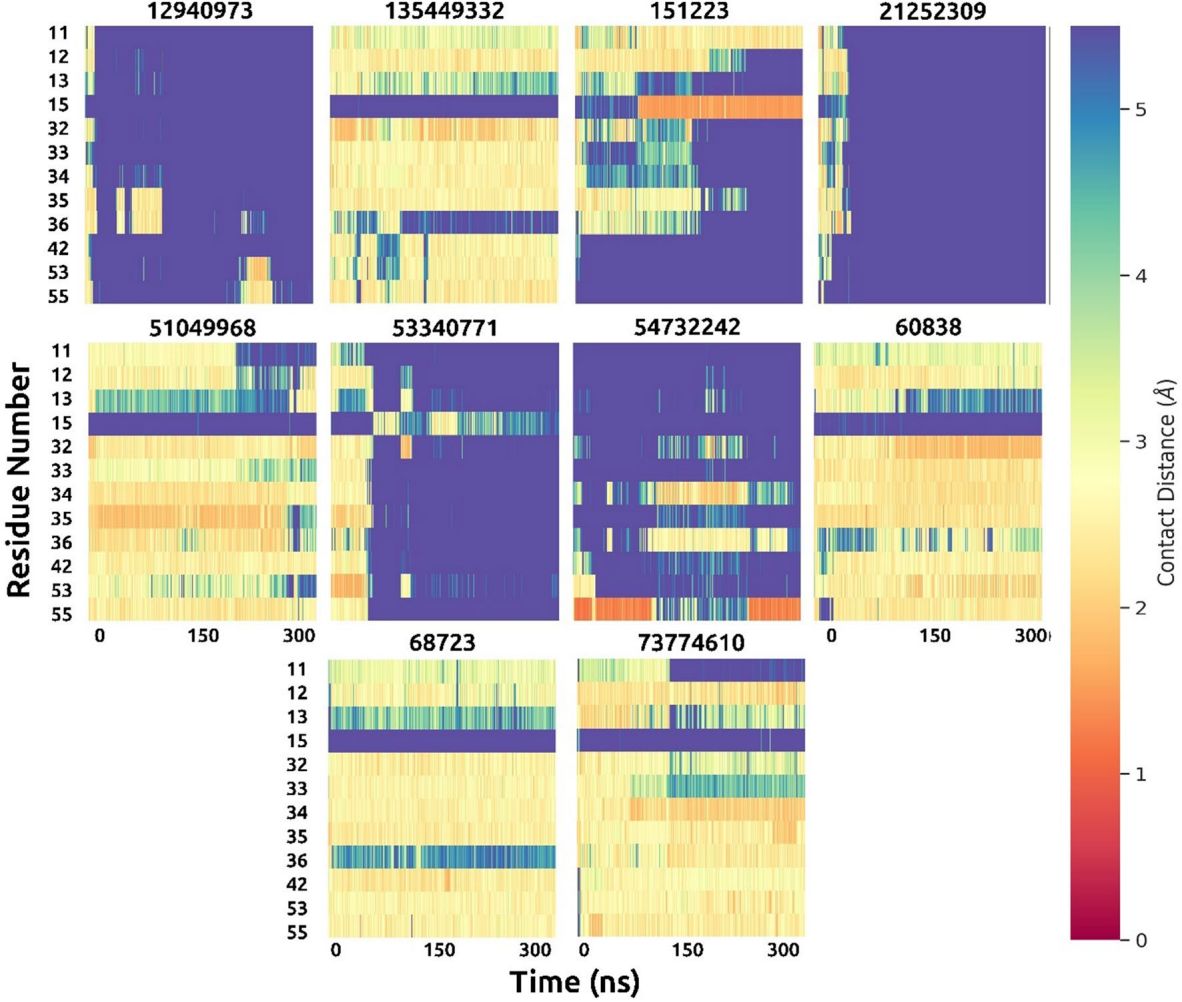
Contact heatmaps of 10 hit compounds during 300 ns simulations. The contact distance is scaled between 0 and 5.5 Å. The warm colors (red to yellow) indicate closer contacts, whereas cold colors (green to violet) indicate farther distance, relatively

The plots of contact heatmaps of the ligands and 2SHP show the close interactions between the ligands and all amino acids in the phosphotyrosine binding site (Fig. 6). CID 60838 shows very close interaction with all amino acids in the binding site. The contact heatmap of CID 60838 shows that this ligand has demonstrated a tight interaction (1–1.5 Å) with Arg32 from 100 to 300 ns of the simulation. Other close interactions were observed with CID 51049968, CID 135449332, CID 68723 and CID 73774610. The interaction distances of other ligands were observed longer than the interactions of CID 60838. These results are in line with the findings in Fig. 3A in which the distance between COM of ligands and COM of Arg32. All ligands which have a closer distance with COM of Arg32, have closer contacts with all amino acids in the target phosphotyrosine binding site.

There are a lot of interactions to stabilize the ligand–protein complex including hydrogen bonds (H-bond), hydrophobic interactions, and electrostatic interactions. H-bond formation is especially crucial for the formation of more stable complexes. H-bond formation was investigated during the MD simulation and hydrogen bond plots [number of the H-bonds vs. time (ns)] were demonstrated (Figs. S1–S3). Hydrogen bond plots show that all ligands have formed H-bonds with the target amino acids. These results were correlated with the molecular docking data (see Table 1 and Fig. 1). As mentioned before, all 10 hit ligands were able to make H-bonds. However, it was observed that some of the ligands lost their H-bond-making ability, or they could not make stable H-bonds during the simulation. According to H-bond analysis, the number of the H-bonds between the following ligands and protein decreased; CID 53340771, CID 21252309, and CID 12940973. After 100 ns of the simulation, the number of the H-bonds increased for the ligands CID 60838, CID 135449332, and CID 151223. In particular, CID 151223 formed 6 stable H bonds for the last 75 ns of the simulations.

**Fig. 7:**
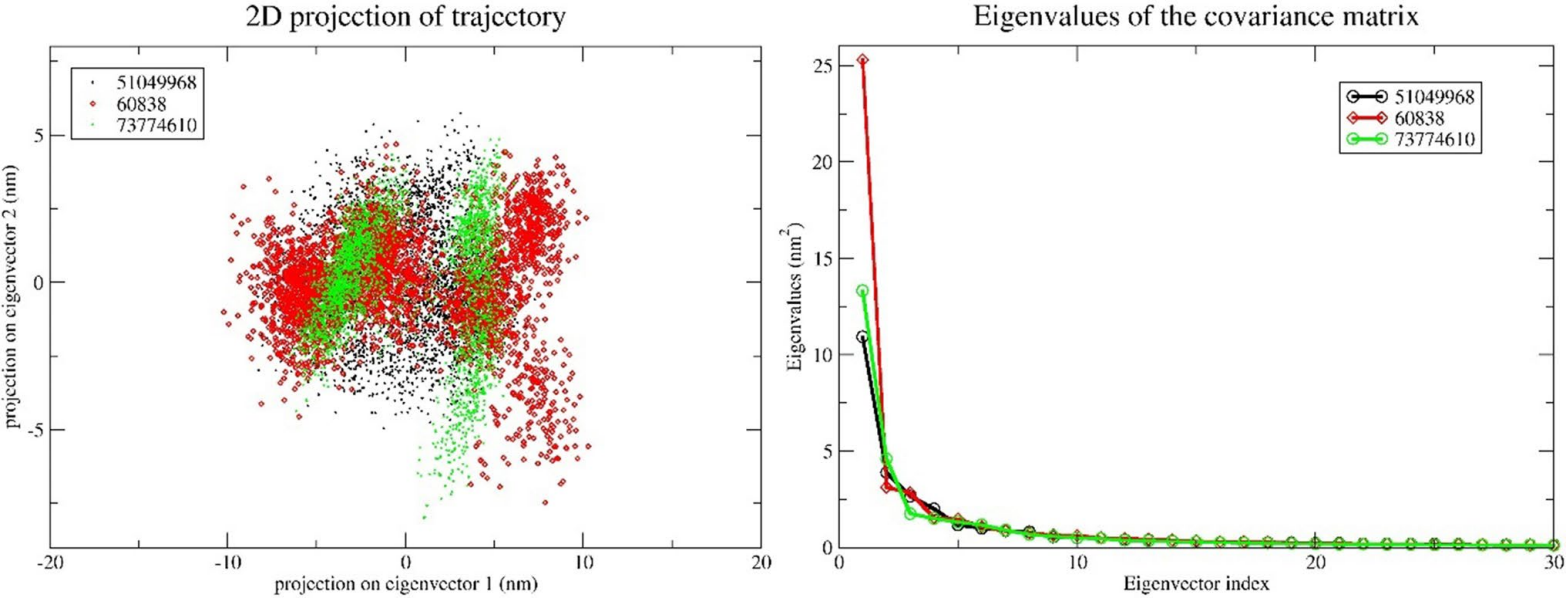
Principal component analysis of top three protein–ligand complexes. The collective motion of CID 60838/protein (red), 7CID 3774610/protein (green), and CID 51049968 (black) using projections of MD trajectories on two eigenvectors corresponding to the first two principal components

Principal component analysis (PCA) was performed on the backbone atoms of the protein–ligand complexes to explore their conformational flexibility and the variety of conformations sampled during the 300 ns molecular dynamics simulations. The analysis focused on first eigenvectors of the top three ligands. The results are visualized in Fig. 7, which shows a 2D projection of the first two principal components.

To understand the protein's conformational changes, we performed a detailed analysis of its backbone motion throughout the simulations. We analyzed backbone motion for a deeper understanding of conformational dynamics in the simulations. This involved extracting the reference structure of the backbone from the first frame of each trajectory. This allowed us to track the relative motion of the backbone throughout the simulation for each complex. We then employed the complete trajectory data, encompassing atomic positions at each simulated time step, to calculate a covariance matrix. This matrix captures the coordinated motions of the backbone atoms, revealing the statistical relationships between individual atomic fluctuations. In simpler terms, the covariance matrix provides insights into how different backbone segments move together during the simulation. Building upon this information, we calculated the principal components (PCs) of the backbone motion. These PCs effectively identify the directions of greatest mobility. Finally, the trajectory was projected onto the top two PCs, resulting in a simplified 2D representation of the backbone's movement over time, as depicted in Fig. 7.

As evident in Fig. 7, the CID 60838/protein complex occupies a larger area in the PCA plot compared to the other complexes. This observation aligns well with the detailed analysis of the backbone motion. The larger area signifies a higher diversity of conformations explored by the CID 60838 ligand during the simulation. Conversely, the tighter clustering of the other complexes suggests a more restricted conformational space. This implies that the CID 60838 ligand exhibits greater flexibility within the binding pocket, while the other ligands adopt more well-defined conformations. Interestingly, this increased flexibility of the CID 60838 ligand might be a contributing factor to its well-stabilized interaction with the protein throughout the simulation. The ability to sample a wider range of conformations could allow the ligand to form favorable interactions with different residues within the binding pocket, distributing the energetic burden and enhancing the overall stability of the complex.

It is worth mentioning that the compound CID 68723 shows why H-bond formation is very important for the stability of the molecules. When we examine the whole molecular docking and MD simulations results of CID 68723; it demonstrates 4 H-bonds with its best docking pose (Table 1), it is the closest ligand to the COM of Arg32 (Fig. 3A), it has very low RMSD values (Fig. 5), and it has remarkable close distance interactions with important residues in the binding site (Fig. 6). However, its binding free energy is higher (− 13.43 kcal/mol) compared to other ligands since it makes a smaller number of H-bonds (Fig. S3) during 300 ns simulations.

## Conclusions

SH2 domains are capable of recognizing phosphorylated tyrosine residues, and thus play important roles in cellular functions. This critical recognition occurs through the very well-conserved universal Arg residue which resides at the βB5 chain of SH2 domains at position 32 [37]. SH2 domains attract attention for targeting in a wide variety of disorders such as cancer, autoimmune diseases, and are potential targets for drug development [38]. The motivation of this study is to target Arg32 specifically to potentially inhibit the protein–protein interactions that occur via this specific residue. For this purpose, we have investigated the potential small molecule inhibitors of the N-SH2 domain of SHP2. After the molecular docking studies of a set of compounds retrieved from Broad Repurposing Hub and ZINC15, we focused on 10 compounds that have the best docking scores among the tested compounds for further MD analysis and binding free energy calculations by MM/PBSA. After all investigations and interpretations of in silico studies, CID 60838 (Irinotecan) has shown the best inhibitory potential against the N-SH2 domain of SHP2. Having a remarkable binding free energy (− 64.45 kcal/mol), especially interacting directly with Arg32 and other residues in the N-SH2 domain during 300 ns MD simulation makes this compound special. In conclusion, although further in vitro and in vivo studies are required, the gathered information in this study might provide a new direction for the design and development of the new inhibitors targeting the N-SH2 domain of SHP2.

## Supplementary Information

Below is the link to the electronic supplementary material.

Supplementary file1 (DOCX 1268 KB)

Supplementary file2 (MP4 33247 KB)

Supplementary file3 (MP4 20949 KB)

Supplementary file4 (MP4 26229 KB)

Supplementary file5 (MP4 24311 KB)

Supplementary file6 (MP4 27686 KB)

Supplementary file7 (MP4 29060 KB)

Supplementary file8 (MP4 30058 KB)

Supplementary file9 (MP4 30617 KB)

Supplementary file10 (MP4 29852 KB)

Supplementary file11 (MP4 25356 KB)
