# Supplementary material for: Determination of promising inhibitors for N-SH2 domain of SHP2 tyrosine phosphatase: an in silico study
Source: Mol Divers. 2024 May 13;28(5):3393–407. doi: 10.1007/s11030-024-10880-2 (PMC11612003; doi:10.1007/s11030-024-10880-2)
Supplement: Supplementary file 1 — Supplementary file1 (DOCX 1268 KB) [file 11030_2024_10880_MOESM1_ESM.docx]

*Supporting Information*

**Determination of Promising Inhibitors of N-SH2 Domain of SHP2 tyrosine phosphatase: An *in silico* study**

Emel Başak Gencer Akçok^a^, Hüseyin Güner^a^, İsmail Akçok^b*^

*^a^Faculty of Life and Natural Sciences, Department of Molecular Biology and Genetics, Abdullah Gül University, 38080, Kayseri, Turkiye*

*^b^Faculty of Life and Natural Sciences, Department of Bioengineering, Abdullah Gül University, 38080, Kayseri, Turkiye*

* Corresponding author: [ismail.akcok@agu.edu.tr](mailto:ismail.akcok@agu.edu.tr)

**Table of Content:**

| **Content** | **Page** |
| --- | --- |
| Molecular Dynamics (MD) methodology | 2 |
| **Table S1:** MM/PBSA Binding Free Energies of Ten Hit Ligands | 3 |
| **Table S2:** Binding Free Energy calculations values | 4 |
| **Figure S1**: H-bonds plots of CID 12940973, CID 135449332, CID 151223 and CID 21252309. | 5 |
| **Figure S2**: H-bonds plots of CID 51049968, CID 53340771, CID 54732242 and CID 60838. | 6 |
| **Figure S3**: H-bonds plots of CID 68723 and CID 73774610. | 7 |
| **Figure S4:** Superposition of frames of selected ligands collected during MD simulations. | 7 |
| **Figure S5:** RMSF of top 3 ligands (60838, 51049968 and 73774610) | 8 |
| **Figure S6:** Radius of gyration calculated during the 300 ns MD simulations | 8 |

**Molecular Dynamics (MD) methodology**

MD simulations were carried out using Gromacs ver. 2021.03^1^. The protein structure was cleaned and prepared for running simulations processing the script provided by the Chimera package. OPLS-AA/M as the force field and SPC216 as the explicit water models were selected to describe the ensemble. Ligand topologies and their force field parameter files for the force field selected were calculated by LigParGen^2^ command-line tool. To initiate a comprehensive series of molecular dynamics (MD) simulations, the initial step involved the energy minimization of the protein-ligand complex along with the surrounding solvent and ion molecules. This process was essential to attain a stable starting configuration for subsequent simulations. The steepest descent method was employed for energy minimization, executed over a maximum of 50,000 steps. The minimization process was halted if the maximum force on any atom reached 10.0 kJ/mol, ensuring the optimization of the system while preventing excessive perturbations.

Following the energy minimization, the ensemble system underwent equilibration with fixations on pressure and temperature^3^. This step aimed to bring the system to a balanced state before the main MD simulations. The equilibration process involved the application of constraints to control both temperature and pressure, setting the stage for subsequent dynamic simulations under controlled conditions.

Temperature coupling in both the NVT (constant Number of particles, Volume, and Temperature) and NPT (constant Number of particles, Pressure, and Temperature) ensembles was achieved through the implementation of the modified Berendsen thermostat^4^. Specifically, the V-rescale method was employed to regulate the temperature throughout the simulations. This approach ensures a controlled and gradual adjustment of the system temperature, contributing to the stability of the simulated dynamics.

For the NPT ensemble, an additional coupling method was introduced to control the system's pressure. The Berendsen pressure coupling technique was selected for this purpose. This method maintains a stable pressure by scaling atomic velocities in response to deviations from the target pressure. The combination of the V-rescale thermostat for temperature control in both NVT and NPT ensembles, along with the Berendsen pressure coupling exclusively for the NPT ensemble, aimed to achieve a balanced and realistic representation of the system's thermodynamic properties during the molecular dynamics simulations.

The primary molecular dynamics (MD) simulation, conducted for a total duration of 300 nanoseconds, utilized the leap-frog integration method with a time interval of 2 femtoseconds. This extensive simulation comprised a remarkable 150 billion steps to capture a comprehensive exploration of the system's dynamics.

Trajectory recordings were made at regular intervals, with snapshots saved every 50,000 steps, allowing for detailed analysis of the system's evolution. Concurrently, energy calculations were performed periodically throughout the simulation, providing insight into the energetic aspects of the molecular dynamics.

The simulation was conducted under three-dimensional periodic boundary conditions, an essential aspect to mimic an infinite system and prevent edge effects. The Parrinello-Rahman method was employed for pressure coupling, ensuring the maintenance of a stable pressure throughout the simulation. Additionally, temperature coupling was achieved through a modified version of the Berendsen method, contributing to the precise control of the system's temperature.

This robust combination of techniques, including the leap-frog integration method, trajectory recordings, periodic boundary conditions, Parrinello-Rahman pressure coupling, and modified Berendsen temperature coupling, collectively formed the core parameters defining the extensive 300-nanosecond MD run and provided a comprehensive exploration of the molecular system under investigation.

**Table S1:** MM/PBSA Binding Free Energies of ten hit ligands

|  | **Ligand** | **Binding energies (kcal/mol)** |
| --- | --- | --- |
| **1** | CID 12940973 | -2,21 |
| **2** | CID 135449332 | -30,33 |
| **3** | CID 151223 | -29,66 |
| **4** | CID 21252309 | 1974,72 |
| **5** | CID 51049968 | -42,13 |
| **6** | CID 53340771 | 0,39 |
| **7** | CID 54732242 | 0,70 |
| **8** | CID 60838 | -64,45 |
| **9** | CID 68723 | -13,43 |
| **10** | CID 73774610 | -33,59 |

**Table S2:** Binding Free Energy calculations values

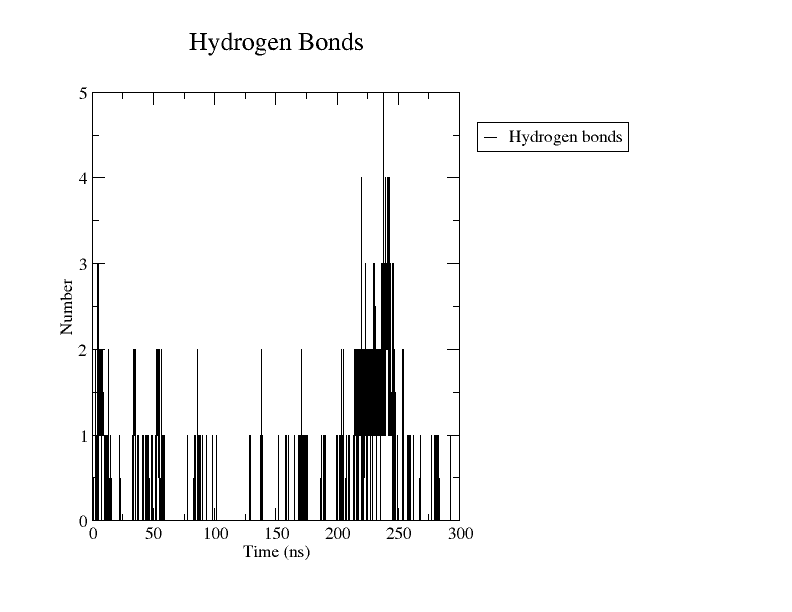

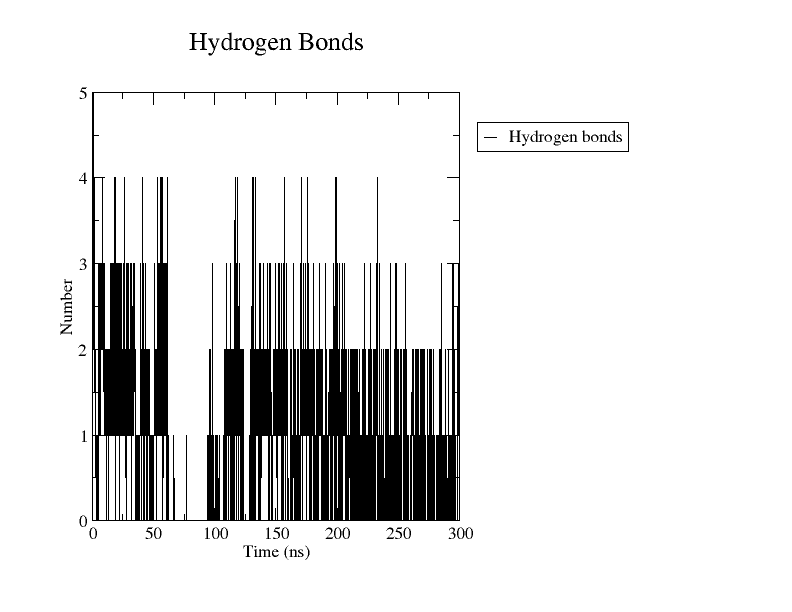


**12940973**

**135449332**


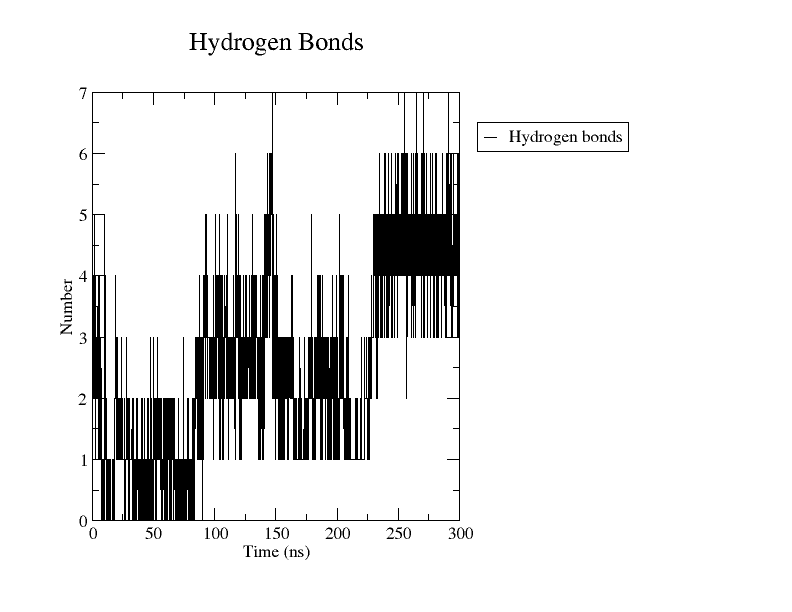

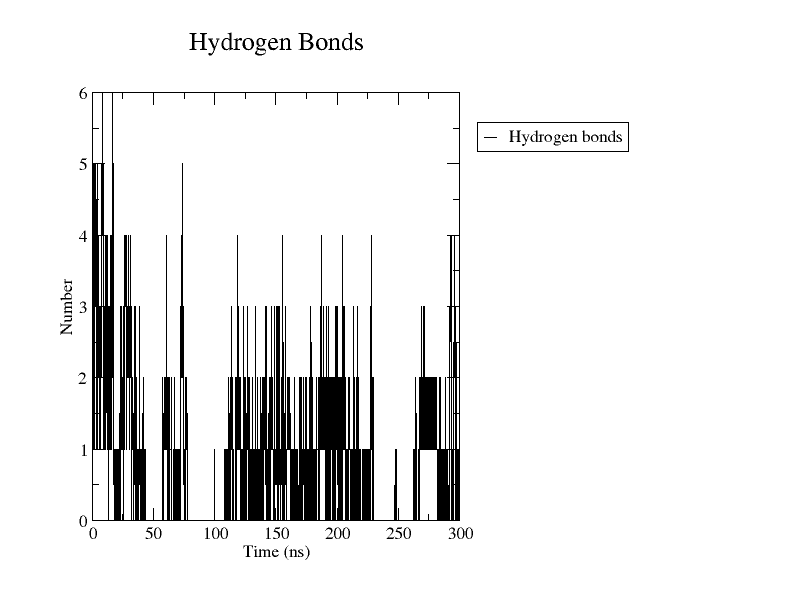


**151223**

**21252309**

**Figure S1**: H-bonds plots of CID 12940973, CID 135449332, CID 151223 and CID 21252309.


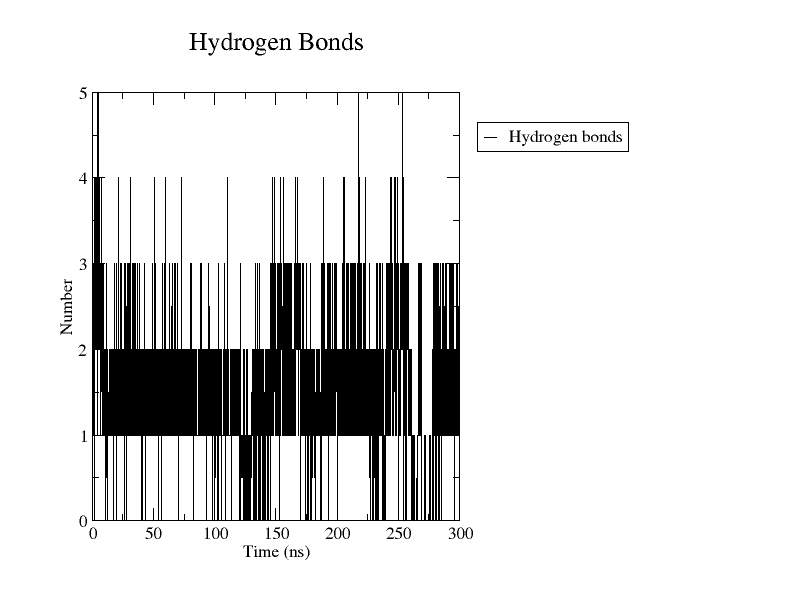

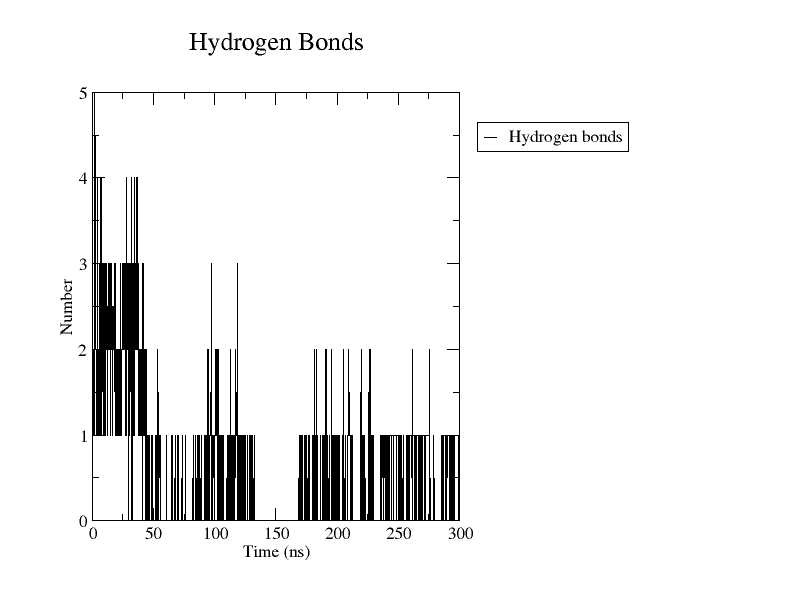


**53340771**

**51049968**


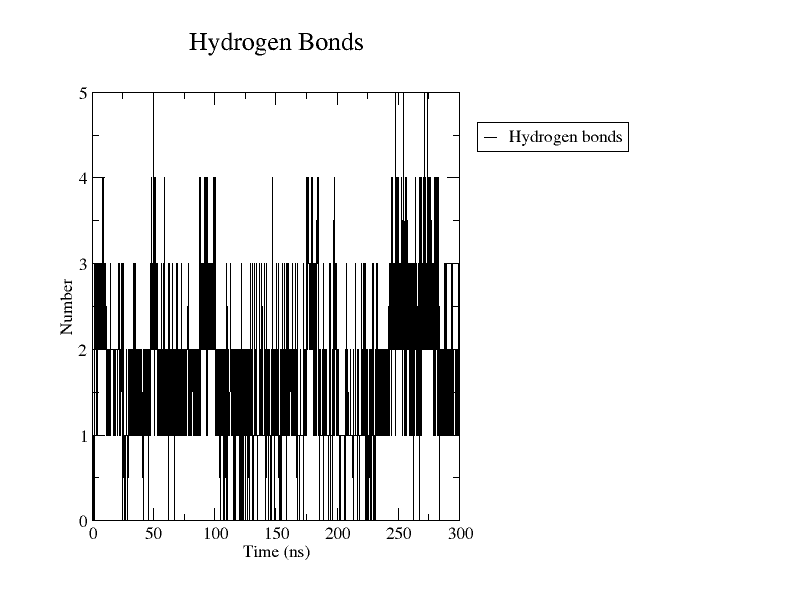

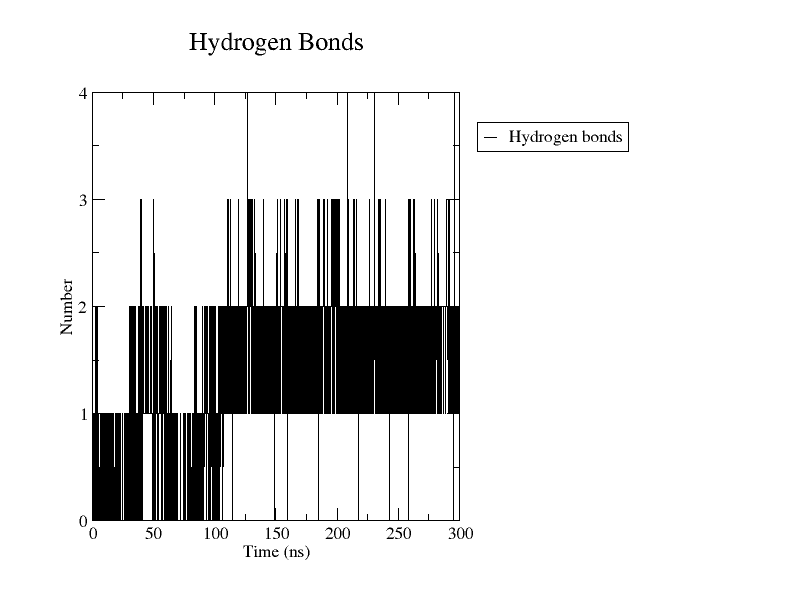


**60838**

**54732242**

**Figure S2**: H-bonds plots of CID 51049968, CID 53340771, CID 54732242 and CID 60838.


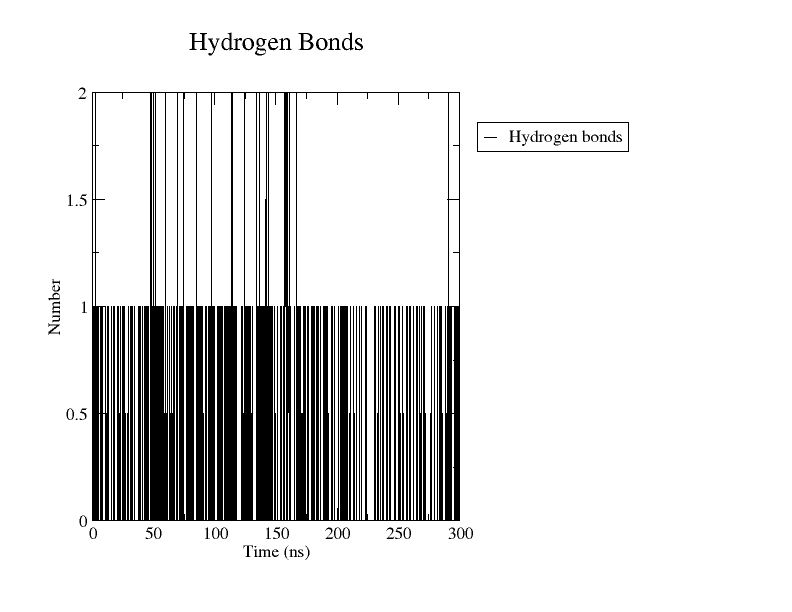

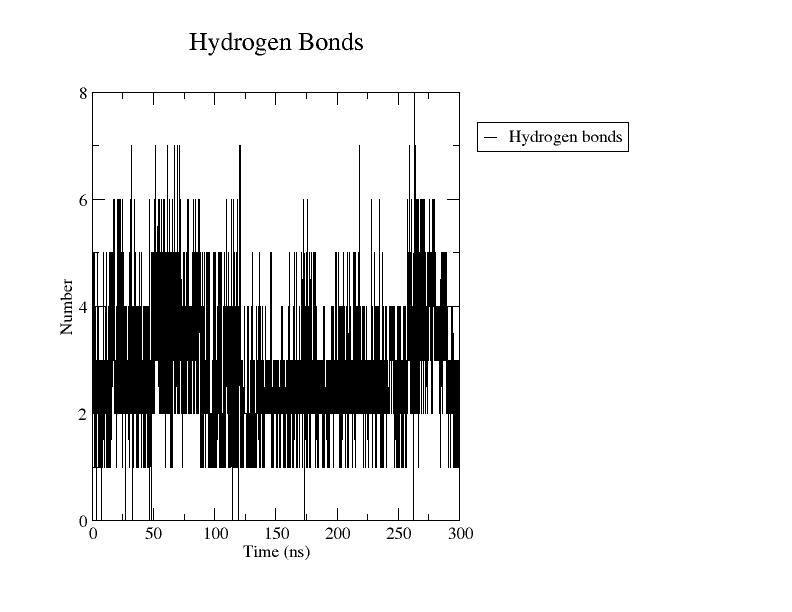


**73774610**

**68723**

**Figure S3**: H-bonds plots of CID 68723 and CID 73774610.

| 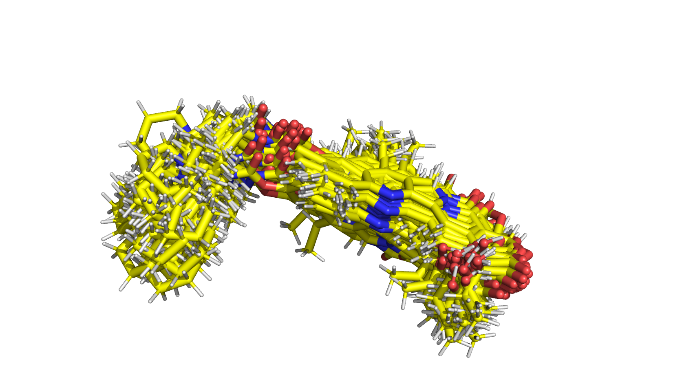  **60838** | 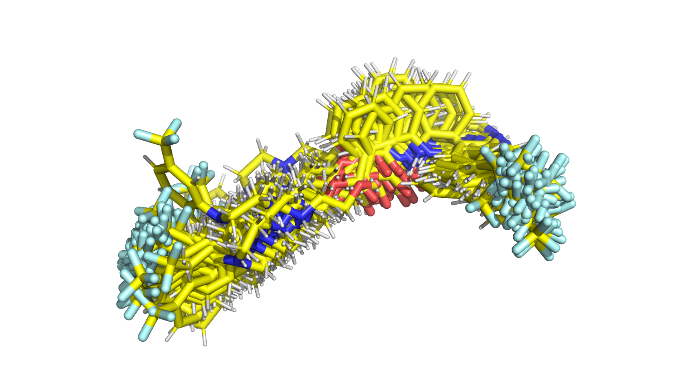  **68723** |
| --- | --- |
| 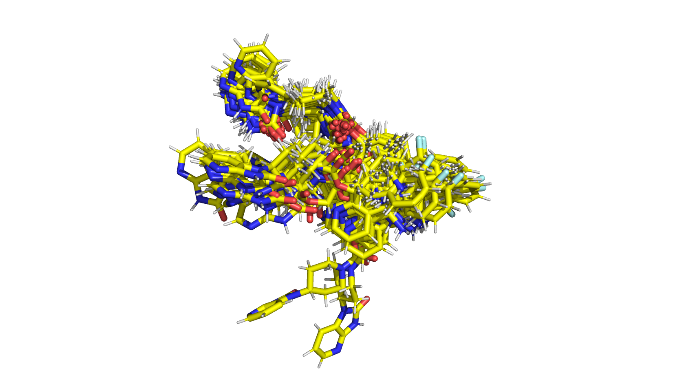  **51049968** | 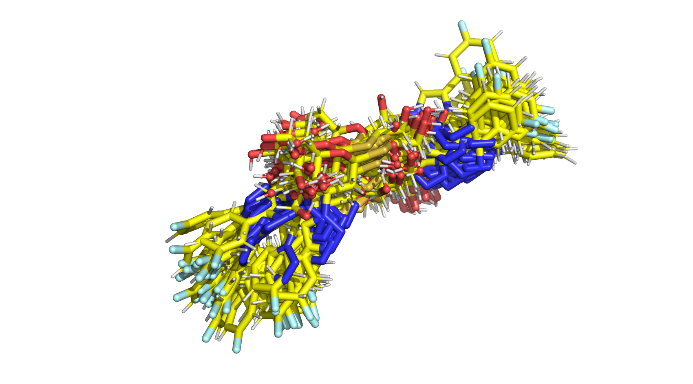  **73774610** |
| 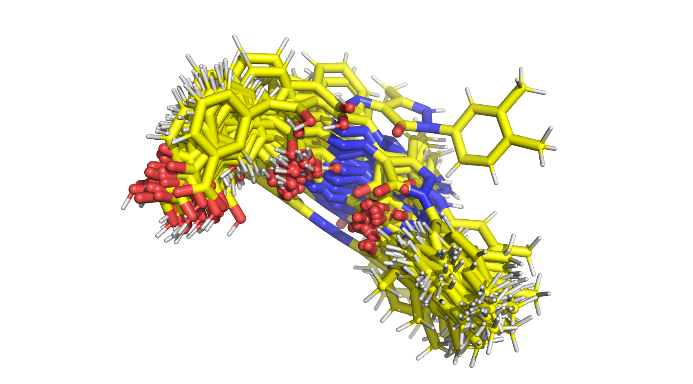  **135449332** | |

**Figure S4:** Superposition of frames of selected ligands collected during MD simulations.


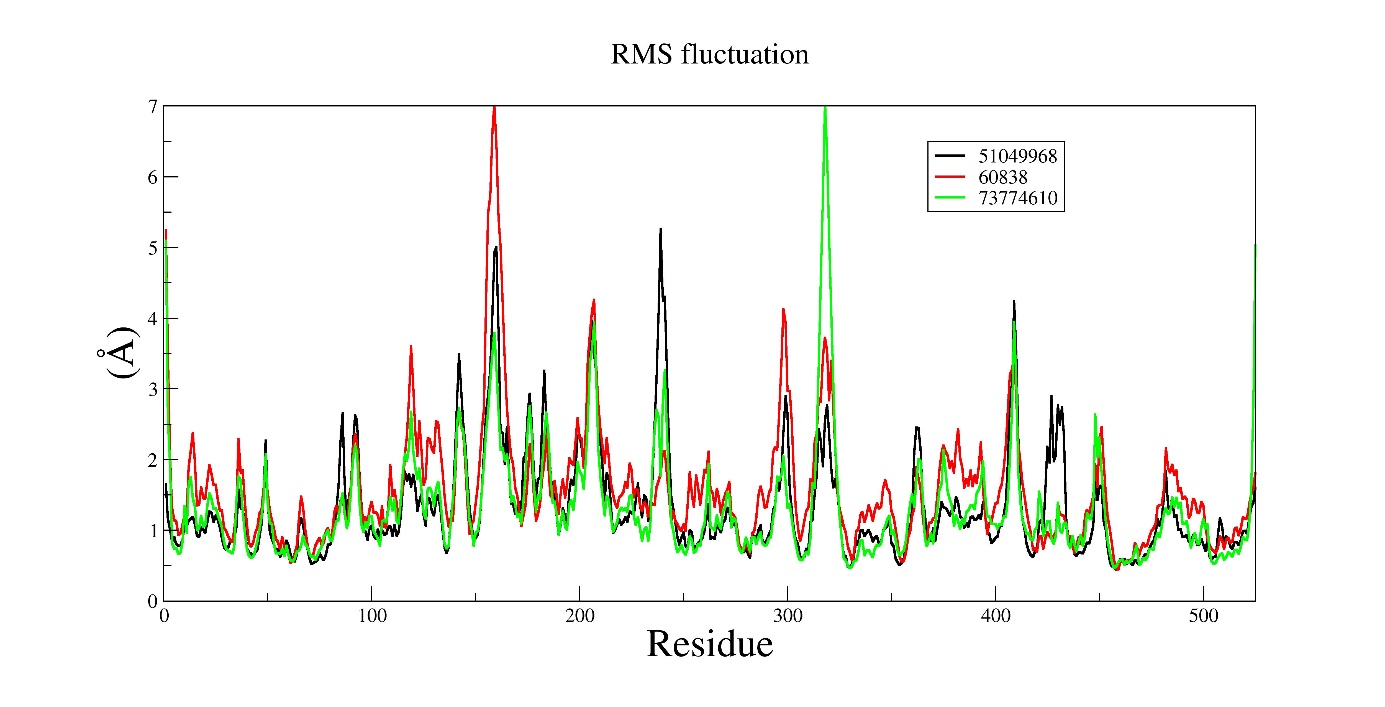


**Figure S5:** RMSF of top 3 ligands (60838, 51049968 and 73774610)


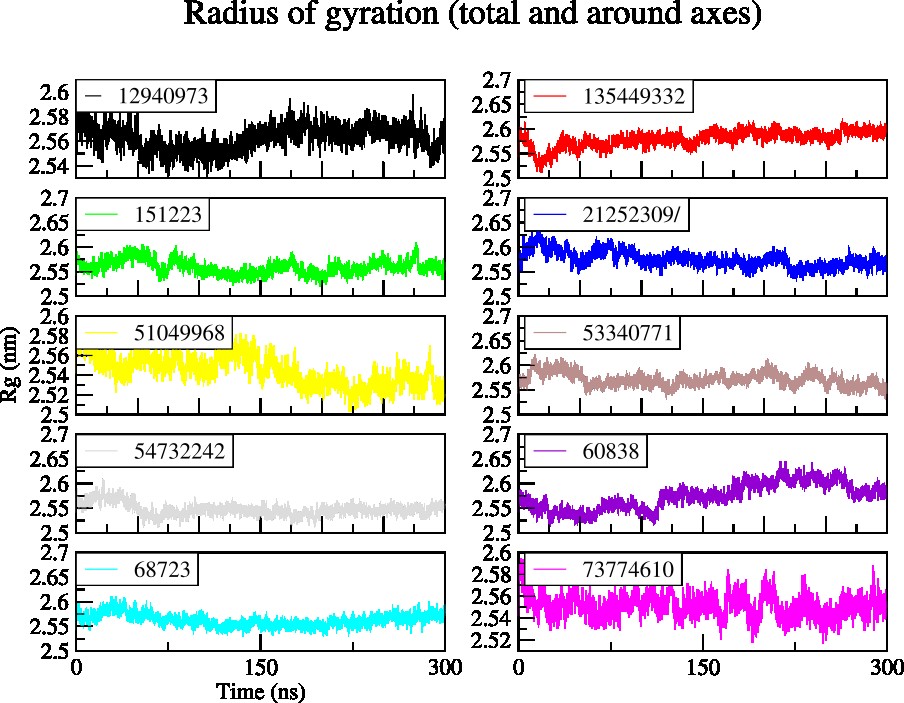


**Figure S6:** Radius of gyration calculated during the 300 ns MD simulations

**References:**

(1) Abraham, M. J.; Murtola, T.; Schulz, R.; Páll, S.; Smith, J. C.; Hess, B.; Lindah, E. Gromacs: High Performance Molecular Simulations through Multi-Level Parallelism from Laptops to Supercomputers. *SoftwareX* **2015**, *1–2*. https://doi.org/10.1016/j.softx.2015.06.001.

(2) Dodda, L. S.; De Vaca, I. C.; Tirado-Rives, J.; Jorgensen, W. L. LigParGen Web Server: An Automatic OPLS-AA Parameter Generator for Organic Ligands. *Nucleic Acids Res.* **2017**, *45* (W1). https://doi.org/10.1093/nar/gkx312.

(3) Van Der Spoel, D.; Lindahl, E.; Hess, B.; Groenhof, G.; Mark, A. E.; Berendsen, H. J. C. GROMACS: Fast, Flexible, and Free. *J. Comput. Chem*. **2005** *26* (16), 1701-1718. https://doi.org/10.1002/jcc.20291.

(4) Berendsen, H. J. C.; Postma, J. P. M.; Van Gunsteren, W. F.; Dinola, A.; Haak, J. R. Molecular Dynamics with Coupling to an External Bath. *J. Chem. Phys.* **1984**, *81* (8), 3684-3690. https://doi.org/10.1063/1.448118.
